# Dissecting the old Mediterranean durum wheat genetic architecture for phenology, biomass and yield formation by association mapping and QTL meta-analysis

**DOI:** 10.1371/journal.pone.0178290

**Published:** 2017-05-25

**Authors:** Jose Miguel Soriano, Marcos Malosetti, Martina Roselló, Mark Earl Sorrells, Conxita Royo

**Affiliations:** 1 Field Crops Programme, IRTA (*Institute for Food and Agricultural Research and Technology*), Lleida, Spain; 2 Biometrics, Wageningen University and Research Centre, Wageningen, The Netherlands; 3 Department of Plant Breeding and Genetics, Cornell University, Ithaca, NY, United States of America; Università Politecnica delle Marche, ITALY

## Abstract

Association mapping was used to identify genome regions affecting yield formation, crop phenology and crop biomass in a collection of 172 durum wheat landraces representative of the genetic diversity of ancient local durum varieties from the Mediterranean Basin. The collection was genotyped with 1,149 DArT markers and phenotyped in Spanish northern and southern locations during three years. A total of 245 significant marker trait associations (MTAs) (*P*<0.01) were detected. Some of these associations confirmed previously identified quantitative trait loci (QTL) and/or candidate genes, and others are reported for the first time here. Eighty-six MTAs corresponded with yield and yield component traits, 70 to phenology and 89 to biomass production. Twelve genomic regions harbouring stable MTAs (significant in three or more environments) were identified, while five and two regions showed specific MTAs for northern and southern environments, respectively. Sixty per cent of MTAs were located on the B genome and 29% on the A genome. The marker wPt-9859 was detected in 12 MTAs, associated with six traits in four environments and the mean across years. To refine QTL positions, a meta-analysis was performed. A total of 477 unique QTLs were projected onto a durum wheat consensus map and were condensed to 71 meta-QTLs and left 13 QTLs as singletons. Sixty-one percent of QTLs explained less than 10% of the phenotypic variance confirming the high genetic complexity of the traits analysed.

## Introduction

Durum wheat (*Triticum turgidum* L. var. *durum*) is grown on around 17 million hectares worldwide. It is a major crop in the Mediterranean Basin, which is the largest durum producing area worldwide, the most significant durum import market and the largest consumer of durum wheat products. Wheat was domesticated in the Fertile Crescent (10,000 BP), and spread to the west of the Mediterranean Basin reaching the Iberian Peninsula around 7,000 years BP [1]. Natural and human selection occurring during this migration resulted in the establishment of local landraces specifically adapted to a diversity of agro-ecological zones, and is considered the largest source of biodiversity within the species [2]. The cultivation of local landraces was progressively abandoned from the early 1970s due to its replacement with the improved, more productive and genetically uniform semi-dwarf cultivars derived from the Green Revolution. However, evidence supports the hypothesis that landraces can provide new alleles for the improvement of commercially valuable traits [3]. Mediterranean landraces represent an important group of genetic resources because of their genetic diversity, their documented resilience to abiotic stresses and their resistance to pests and diseases [3].

In the Mediterranean basin wheat is mainly grown under rainfed conditions and yield is often constrained by water and heat stress that are common during the grain filling period, due to the low and unpredictable seasonal rainfalls and high temperatures during the last stages of the crop season [4]. Moreover, according to the intergovernmental panel for climate change (http://www.ipcc.ch/), drought conditions are expected to worsen, with warmer temperatures and lower and more erratic water availability affecting the major wheat producing areas, including the Mediterranean Basin. In a context of climate change, improving the knowledge of yield and the most important traits underlying the adaptive response of durum wheat to Mediterranean environments is essential to enhance the development of varieties adapted to sub-optimal environments. Exploiting genetic diversity from local landraces in breeding programmes is important for adaptation to harsh environments and end-product quality, given the high level of polymorphism found between and within Mediterranean landraces for traits of commercial importance [5]. Thus, unravelling the genetic mechanisms underlying development, growth and yield formation, under water limited conditions is one of the major challenges for wheat production worldwide.

Association mapping (AM) and linkage Quantitative Trait Loci (QTL) mapping are the two main approaches used to dissect the genetic basis of complex traits [6]. AM is used as a complementary approach to linkage analysis providing broader allelic coverage and offering higher mapping resolution. Association mapping is based on linkage disequilibrium (LD), defined as the non-random association of alleles at different loci, and is used to detect the relationship between phenotypic variation and genetic polymorphisms [7]. It is important, however, to differentiate the LD due to physical linkage from LD due to population structure that can be caused by selection, genetic drift and species-dependent characters such as the mating system [7]. Germplasm collections characterized by medium to high LD levels are suitable for the identification of chromosome regions harbouring genes/QTL controlling agronomic traits in wheat [8]. To date AM has been carried out in many crops. In wheat AM has been recently conducted for investigating the genetic basis of yield and yield components in a number of environments [6, 9–15], grain quality traits [15,16], root traits [17], biomass for bioethanol production [12], resistance to diseases [18], and crop phenology [6, 14].

To synthesize the large amount of information currently available on QTLs for relevant agronomic traits in wheat and its integration on a consensus map is essential in order to accelerate the breeding programmes. One way to synthesize and integrate all QTL information is the QTL meta-analysis approach developed by [19]. QTL meta-analyses have been performed in the last years mostly in bread wheat for grain traits [20], plant height [21], sprouting tolerance and dormancy [22], dietary fiber content in grain [23], grain yield [24], crop phenology [25], and resistance to septoria tritici blotch [26], powdery mildew [27], fusarium head blight [28–30], leaf rust [31] and Ug99 stem rust [32]. However, studies in durum wheat are scarce.

In this study, AM was used to identify genome regions affecting yield formation, crop phenology and crop biomass in a collection of 172 durum wheat landraces representative of the variability existing in the species within the Mediterranean Basin. The population structure of the collection was previously analysed [5] showing a strong eastern-western geographical structure consistently matching with the spread of wheat across the Mediterranean Basin. Additionally, QTL meta-analysis was conducted in the current study to narrow down the QTL intervals and to identify consensus QTL regions controlling the target traits.

## Materials and methods

### Plant material

This study evaluated a collection of 172 durum wheat landraces and old varieties from 21 Mediterranean countries, whose genetic structure and agronomic performance have been recently reported [5]. To represent the genetic diversity of ancient local durum varieties from the Mediterranean Basin, landraces were selected as described in [2]. Seeds provided by public gene banks (Centro de Recursos Fitogenéticos INIA-Spain, ICARDA Germplasm Bank and USDA Germplasm Bank) were bulk-planted and one spike of the dominant type from each bulk (usually with a frequency above 80% of the bulk) was planted on a row the next crop season for purification. The harvested seeds were further increased in plots, ensuring that the seeds used for each experiment had a common origin.

### Phenotyping

Field experiments were carried out during three harvesting seasons (2007, 2008 and 2009) in two locations in Spain (Lleida in the north-east of Spain, referred to as North and Granada, in the south of Spain, further referred to as South) as reported previously by [5].

Phenology traits were dissected in days from sowing to booting (DSB), days from booting to anthesis (DBA) and grain filling duration (GFD), i.e, days from anthesis to physiological maturity. The traits were determined in each plot considering growth stages (GS) 45, 65 and 87, respectively, of the Zadoks scale [33]. Crop dry weight (CDW g m^-2^) at GS21 (beginning of tillering), GS33 (jointing), GS65 (anthesis) and GS87 (physiological maturity) was calculated for each sample as described by [34]. Plant height (PH, cm), and grain yield (GY, t ha^-1^) and yield components: number of spikes per square metre (NSm^2^), number of grains per square metre (NGm^2^), grain weight (GW, mg) and harvest index (HI) were calculated as described by [5].

### Genotyping

DNA isolation was performed from young leaf samples following the method of Doyle and Doyle [35] and sent to Diversity Arrays Technology (Canberra, Australia) (http://www.diversityarrays.com). The durum wheat PstI/TaqI array v2.0, containing 7600 single DArT clones, was used for genotyping the panel of accessions. Loci were designed as ‘wPt’, ‘rPt’ and ‘tPt’, corresponding to wheat, rye and triticale clones, respectively. Polymorphic alleles were scored as 1 or 0 depending on the positive or negative hybridization.

Eleven hundred and forty-nine markers were used to genotype the 172 wheat accessions. To reduce the risk of false positive marker-trait associations (MTAs), rare alleles (i.e., with frequencies lower than 0.05), accessions with more than 25% of missing values and markers with more than 20% of missing values, were excluded from the analysis.

Markers were ordered according to the consensus map of durum wheat developed by Maccaferri et al. [36]. The consensus map presented several recombination gaps lacking polymorphic markers. Thus, chromosomes 1A, 2A and 3A were represented by two linkage groups.

In order to locate markers not positioned by [36], the ‘Wheat consensus map version 3.0’ downloaded from http://www.diversityarrays.com/sequence-maps was used. Additionally, DArT sequences were downloaded from http://www.diversityarrays.com/dart-map-sequences and blasted against the wheat genome sequence at https://wheat-urgi.versailles.inra.fr/Seq-Repository/Assemblies. Only sequences from significant MTAs were blasted.

### Linkage disequilibrium

Only markers with known map positions were used to perform linkage disequilibrium (LD) analysis with TASSEL 3.0 [37]. LD was estimated as the square of marker correlations (*r*^*2*^). Two significance levels were chosen, moderate, *P*<0.01 and highly significant, *P*<0.001. The LD was calculated separately for loci on the same chromosome (intra-chromosomal pairs) and for unlinked loci (inter-chromosomal pairs). A critical *r*^*2*^ value beyond which LD is most likely due to linkage was determined taking the 95^th^ percentile of the root transformed *r*^*2*^ values of unlinked markers [8]. The intra-chromosomal *r*^*2*^-values were plotted against the genetic distance and a LOESS curve was fitted to the plot using Genstat v.18 software (VSN International), to determine at which distance the curve intercepts the line of the critical *r*^*2*^ in order to estimate how fast the LD decay occurs.

### Association mapping

Association mapping was carried out for the best linear unbiased estimates (BLUEs) using a mixed linear model accounting for genetic relatedness using the kinship matrix, the K model implemented in GenStat v.18 (VSN International). The significance of each marker trait association (MTA) was assessed with the Wald test, and results were expressed with the associated *P-*values on a–log_10_ scale.

Graphical representation of the genetic position of MTAs was carried out using MapChart 2.3 [38].

### QTL meta-analysis

Four hundred and seventy-six QTLs were reported in the 26 published studies examined. A total number of 25 traits were studied: CDW_21_, DSH, DSA, DSM, GD, GFD, GW, GWS, GY, HI, LG, MG, NDVI_g_, NDVI_v_, NFs, NFTm^2^, NGm^2^, NGS, NSP, NsS, NSm^2^, NTP, PH, SG and SL.

For each study, the following information was collected: parents of the cross, type of cross, number of progenies, name of QTLs, trait, environment, LOD score, PVE (phenotypic variance explained) by each QTL, QTL position on the authors’ linkage map, flanking markers and QTL supporting interval (SI).

To compare the QTLs detected in different populations, original QTL data were projected onto the consensus map of durum wheat developed by [36]. QTLs were projected following the homothetic approach proposed by Chardon et al. [39]. The SI were defined as reported by [31] and estimated at 95% on the consensus map using the empirical formula proposed by [40] and [41]:

SI = 163/(*N* x *R*^*2*^) for recombinant inbred line (RIL).
SI = 530/(*N* x *R*^*2*^) for doubled haploid (DH), backcrosses (BC) and F_2_ progenies.

Where *N* is the size of the population and *R*^*2*^ the proportion of variance explained by the QTL.

QTL meta-analysis was conducted following the approach of Goffinet and Gerber [19] and Veyrieras et al. [42] using BioMercator v4.2 [43, 44], available at http://moulon.inra.fr/. Additionally, MQTLs reported previously by [24] for yield components and [25] for phenology were projected onto the consensus map for further comparisons. Graphical representation of the genetic position of MQTLs was carried out using MapChart 2.3 [38].

## Results

### DArT markers

In order to genotype the 172 durum wheat landraces, 1,149 DArT markers were selected from the durum wheat PstI/TaqI array v2.0 due to their polymorphism. In order to reduce the risk of false positives. markers and accessions were analysed for the presence of duplicated patterns and missing values. Forty six markers with a duplicated scoring pattern in the accessions, five markers with more than 20% missing values and 24 markers with rare alleles (i.e. frequency lower than 5%) were excluded, as well as three accessions with more than 25% missing values. The final genotype file comprised 169 durum wheat landraces, and 1,074 DArT markers.

Markers were ordered according to the consensus map of durum wheat developed by [36]. Eight hundred and forty four markers had a known position in the linkage map, 111 were assigned to a linkage group but their position on it was unknown, and finally 119 were not located in any linkage group. The DArT markers were distributed across the 7 homologous chromosome groups. Chromosomes 1A, 2A and 3A presented two linkage groups. The map covered a length of 2169.4 cM, ranging from 11.6 cM in chromosome 3A (LG1) to 232.7 cM in chromosome 2B. The number of markers per chromosome ranged from 12 in chromosome 5A to 105 in chromosome 6B. As reported by [36] in the consensus map, the number of markers assigned to the A genome (327) was less than those assigned to the B genome (517), 39% and 61% respectively.

### Linkage disequilibrium analysis

Linkage disequilibrium was estimated for locus pairs in the same chromosome (intra-chromosomal loci) and for unlinked loci (inter-chromosomal loci). Using the 172 genotypes with 842 DArT markers with known positions, 606,650 possible pair-wise loci were observed. Of these locus pairs, 15.1% and 7.1% showed significant linkage disequilibrium at *P*<0.01 and *P*<0.001, respectively. There were 60 (5A) to 5355 (6B) possible locus pairs in the 17 linkage groups. The percentage of locus pairs showing significant LD at *P*<0.01 ranged from 13.1% (2B) to 58.9% (2A-LG2) and at *P*<0.001 from 6.8% to 41.2% in the same linkage groups.

The mean *r*^*2*^ for unlinked loci was 0.02, whereas for intra-chromosomal loci ranged from 0.03 (2B, 7A) to 0.43 (1A-LG2). A critical value of *r*^*2*^>0.28 was determined as the threshold for LD due to physical linkage. The decay of LD varied in each chromosome as it is reported in S1 File. Markers were in LD in a range from less than 1cM (chromosomes 3A-LG1 and 5A) to ~ 8cM (chromosomes 3A-LG2 and 3B).

### Association mapping

**Fig 1:**
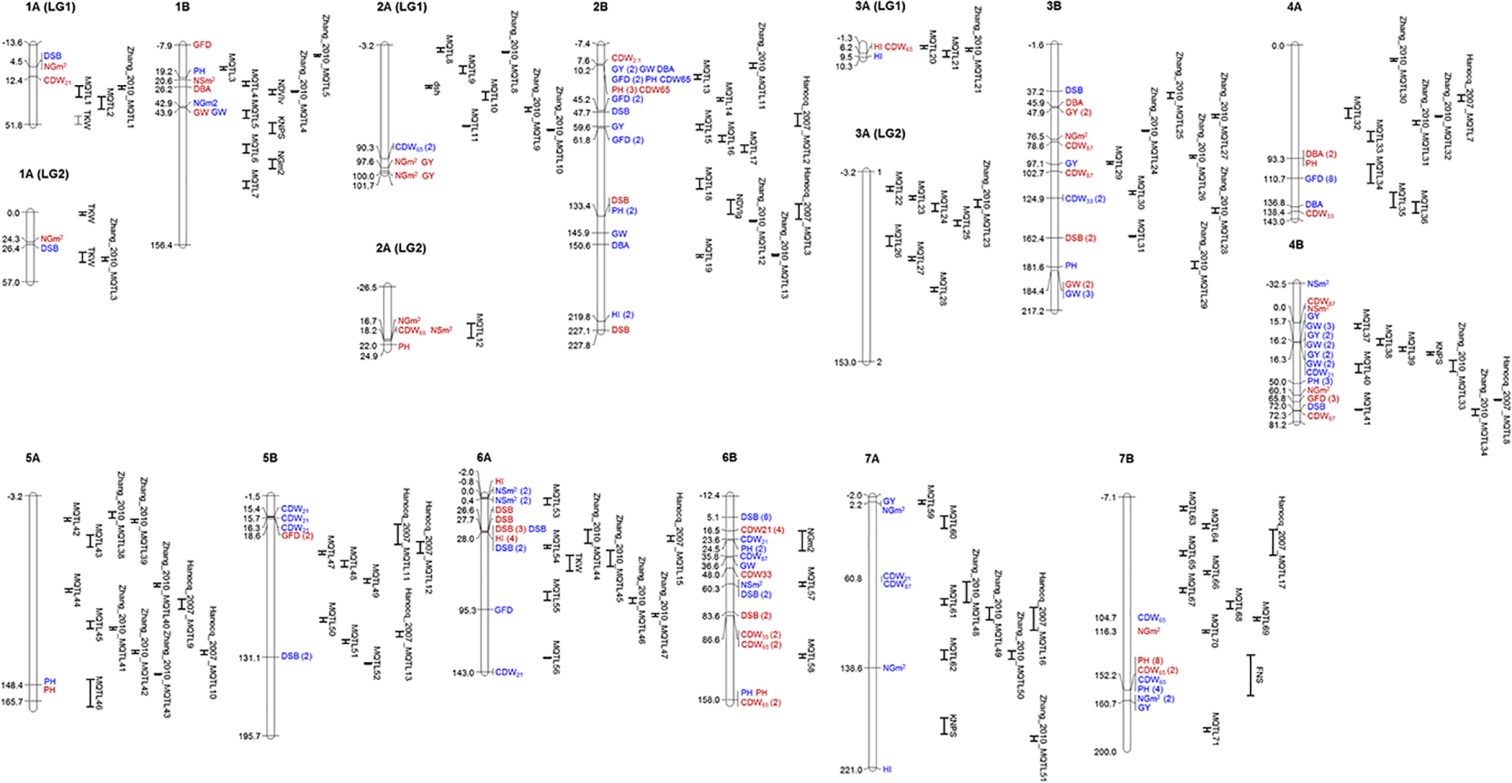
Genetic map for significant MTAs. Genetic position in the durum wheat consensus map from Maccaferri et al. [36] of significant MTAs in the north (blue) and south (red) Spanish locations together with the position of MQTLs reported in this study and by other authors [24, 25]. Numbers in parenthesis after MTAs represent the number of different MTAs for the trait. When a MQTL involved only one QTL, it is reported as the trait for that QTL. For the extended name of the traits, see the list of acronyms.

Association mapping (AM) was performed with 1074 DArT markers. Results of AM are reported in S2 File and Fig 1. Using a threshold of–log_10_(*P*) > 3 a total of 245 marker trait associations (MTAs), involving 154 DArT markers, were detected for the 13 measured or calculated traits in six environments, North (N) and South (S) during 3 years (2007, 2008, 2009) and calculated mean data across environments (NM and SM) [5]. The ‘Wheat consensus map version 3.0’ downloaded from http://www.diversityarrays.com/sequence-maps and the BLAST tool at https://wheat-urgi.versailles.inra.fr/Seq-Repository/Assemblies allowed us to identify the linkage group and the physical position of 2 and 12 previously unmapped MTAs respectively (S2 File).

The MTAs were located in all chromosomes with 66% of MTAs located on genome B and 31% on genome A, leaving 3% of MTAs unassigned to any chromosome. The highest number of MTAs identified were on chromosomes 2B and 6B (30) followed by chromosome 7B with 27 MTAs. On the contrary, the lowest number of MTAs were located on chromosomes 5A (4), 3A (5) and 7A (7).

Eighty-six MTAs corresponded with yield and associated traits (GW, NSm^2^, NGm^2^, HI), 70 to phenology development and 89 to biomass (PH, CDW) (Table 1). Taking into consideration the location latitude, 141 MTAs were detected in the north and 104 in the south. By years, the highest number of significant MTAs (70) were detected in 2007, followed by 2008 and 2009 with 52 and 51 MTAs, respectively. Using the mean data across years, a total of 72 MTAs were detected.

**Table 1:** Number of significant associated markers.

Trait^a^	N7	N8	N9	NM	S7	S8	S9	SM
GY	4	1	2	4	4	1	0	0
NGm^2^	3	1	0	1	3	2	3	0
TKW	3	8	2	7	3	0	1	6
HI	1	1	0	5	0	1	0	6
NSm^2^	1	1	2	2	1	1	4	1
Total yield	12	12	6	19	11	5	8	13
CDW_21_	6	0	1	2	2	0	2	2
CDW_33_	1	1	0	1	1	1	0	2
CDW_65_	0	2	1	3	3	1	6	1
CDW_87_	3	0	1	0	0	1	5	0
PH	5	9	3	5	5	4	4	5
Total biomass	15	12	6	11	11	7	17	10
DSB	6	6	2	8	4	0	3	3
DBA	4	1	0	1	1	2	2	1
GFD	6	3	5	6	0	4	2	0
Total phenology	16	10	7	15	5	6	7	4
**Total**	**43**	**34**	**19**	**45**	**27**	**18**	**32**	**27**

Number of DArT markers associated with the assessed traits at each environment and in the calculated mean across them in north and south.

^a^For the extended name see list of acronyms.

Out of the 154 markers, 69% were detected only in a single environment, and the remaining 31% in two or more environments, including mean data across years. One hundred and twenty-eight markers were associated with only one trait and the remaining 26 markers from two to six traits. A total of 31 MTAs (13%) involved unmapped markers.

Based on the category of the traits, 35% of MTAs detected in the north corresponded to yield and associated traits, 34% with crop phenology and 31% with biomass. In the south the biomass related traits represented the 43% of the MTAs, followed by yield (36%) and phenology (21%).

Among the different regions showing MTAs, twelve of them contained the most stable MTAs, detected in at least three environments (Table 2). Five of these regions showed MTAs involved in yield and related traits, seven reported associations with biomass traits and 5 with phenology development. Chromosome 6B showed the highest number of chromosome regions with stable MTAs (3). The marker wPt-9859 located on chromosome 2B (10.2 cM) was detected in 12 MTAs, associated to six traits in four environments and mean data across years.

**Table 2:** Chromosome regions containing the most stable MTAs.

Chromosome	Position (cM)	Trait^a^	Environments^b^	Effect^c^
1B	42.9–43.9	NGm^2^, GW	N7, NM, S9	-/+
2B	10.2	GY, GW, DBA, GFD, PH, CDW_65_	N7, N8, NM, S7, S9, SM	-/+
2B	133.4	PH, DSB	N7, N8, S9	+
3B	181.6–184.4	PH, GW	N7, N8, N9, NM, S7, SM	-/+
4B	15.7–16.3	GY, GW	N7, N8, NM	-
4B	50.0	PH	N8, N9, NM	+
5B	18.5–25.6	GFD, DBA	S9, N8, NM	+/-
6A	26.6–28.0	DSB, HI	NM, S7, SM	-/+
6B	5.1	DSB	N8, N9, NM	-
6B	16.5–24.7	CDW_21_, PH	N7, N8, S9, SM	+
6B	158	CDW_33_, CDW_65_, PH	N8, S7, S8, SM	+/-
7B	152.2	CDW_65_, PH	N7, N8, NM, S7, S8, S9, SM	-

^a^For the extended name see list of acronyms.

^b^N: north. S: south, followed by a figure indicating the harvesting year M: mean value across the 3 years.

^c^Minor allele effect

Chromosome 1A showed a stable MTA region for NGm^2^ and GW (42.9–43.9 cM) reported in north and south environments. On chromosome 2B the marker wPt-1140 (133.4 cM) showed effects for PH and DSB in north and south environments, respectively. On chromosome 3B a locus controlling PH and GW (181.6–184.4 cM) was detected in both environments reducing PH and increasing GW. Chromosome 4B harboured a locus with effects on yield and GW only significant in the north environment. A second locus on 4B located at 50.0 cM corresponding with the marker wPt-6209 was significant for PH in the north environment. A locus on chromosome 5B increased GFD and decreased DBA in north and south environments, respectively. On chromosome 6A a locus was detected for DSB and HI in the north and the south reducing the number of days from sowing to booting and increasing HI. Chromosome 6B reported the highest number of regions with stable MTAs (3). The first of these regions showed MTAs reducing DSB in the north. The second region was involved in biomass traits, producing a gain in both CDW at tillering and PH. The last region was also involved in biomass traits, increasing CDW at anthesis and reducing PH. Finally, on chromosome 7B another region controlling biomass traits was detected in 3 and 4 environments in the north and the south, respectively. Some of these regions showed other MTAs, but present in less than three environments, thus they were not considered stable for the corresponding traits.

**Fig 2:**
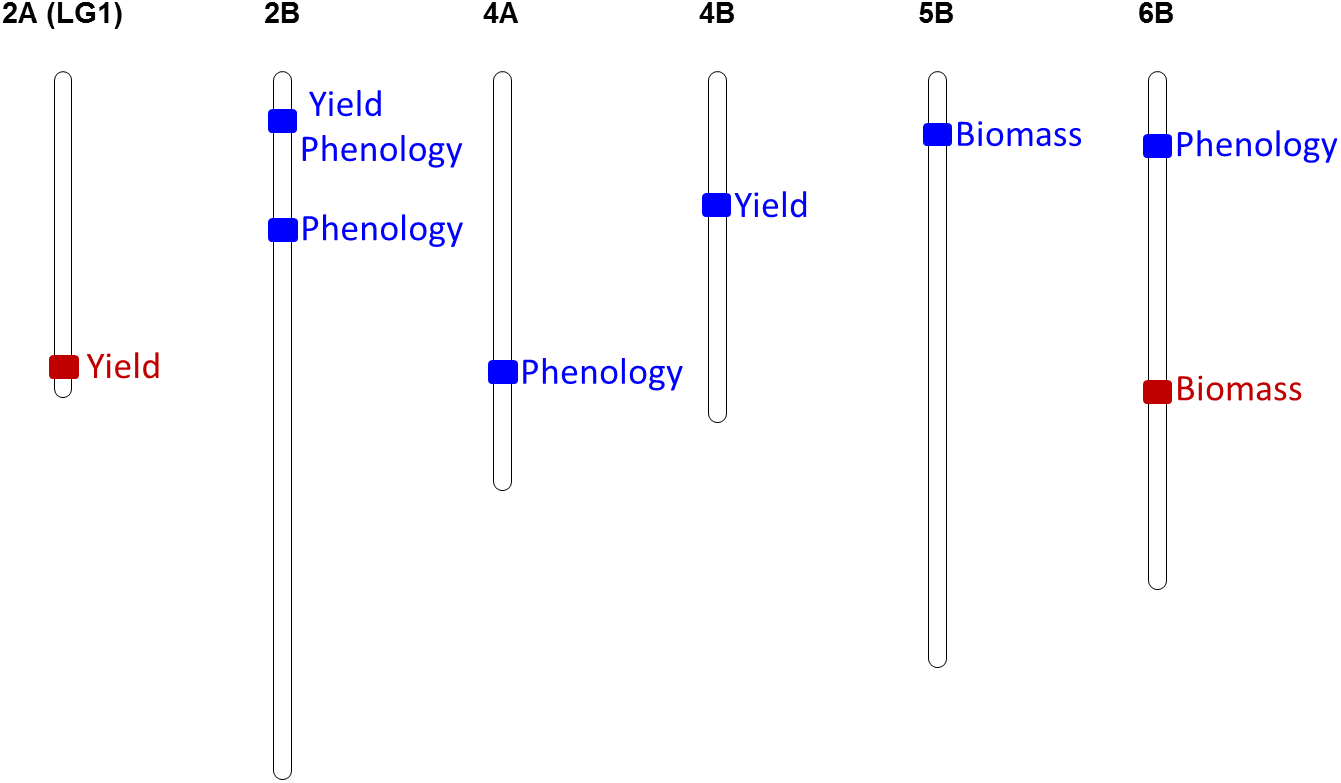
Specific MTAs for north and south. Genomic regions showing specific MTAs for the north (blue) or the south (red).

Although most regions reported MTAs belonging to north and south locations, regions with MTAs specific for each one of them were found (Figs 1 and 2). These regions were considered if they included at least 3 MTAs belonging to the same trait category, yield, biomass or phenology. Six regions were found to be specific from north environment. In four of these regions associations were found corresponding with phenological development, two for yield and one for biomass. In the south no specific regions were found for phenology, and only two were detected, one for yield and one for biomass traits.

### QTL meta-analysis

This survey collected data from 24 studies reporting QTLs for wheat yield and yield components, biomass related traits and phenology, published from 2008 to 2015 (Table 3). The studies covered 23 different experimental crosses involving 44 parental lines and 4438 progenies. The traits were evaluated in a total of 117 environments and 477 QTLs were subjected for QTL projection onto the durum wheat consensus map developed by Maccaferri et al. [36].

**Table 3:** Summary of QTL studies included in the meta-analysis.

Cross	Progeny^a^	Size	Traits^b^	Environments	QTLs^c^	Reference
Louise x Penawawa	RIL	188	PH	5	7	[45]
CDC Teal x CDC Go	RIL	187	DSA, DSH, DSM, FTm^2^	4	15	[46]
Wimai 8 x Jimai 20 (WJ)	RIL	175	NGS, GWS, NSP, GW	4	23	[47]
Wimai 8 x Yannong 19 (WY)	RIL	172	NGS, GW	4	19	[47]
Wimai 8 x Luohan 2 (WL)	RIL	179	NGS, NSP, GW	3	19	[47]
05210 x Laizhou953	F2	166	NGS, NSP	1	2	[48]
Kofa x Svevo	RIL	249	FTm^2^, NGS, NDVI, NGm^2^, GW	1	17	[49]
Nanda2419 x Wangshuibai	RIL	130	GW, NGS, PH, NSP, NTP	4	34	[50]
AC Barrie x Cutler	RIL	177	DSA, DSM	5	9	[51]
Keumkang x Olgeuru	DH	122	GY, NGS, NSm^2^, GW	1	6	[52]
CNN x CNN (WI 3A)	RIL	223	DSA, GY, NGS, NSm^2^, PH, GW	6	25	[53]
Nanda 2419 x Wangshuibai	RIL	230	DSA	4	8	[54]
ND3331 x Zang1871	RIL	217	NGS, GWS, NSP, PH, GW	9	66	[55]
Lang x CSCR6	RIL	82	NGS, PH, GW	1	3	[56]
Kofa x Svevo	RIL	249	DSH	1	3	[57]
Halberd x Karl92	RIL	118	CDW_21_, DSH, DSM, GFD, GY, NGS, NSm^2^, GW	1	14	[58]
Rye selection 111 x Chinese spring	RIL	92	GW	1	6	[59]
HTRI 11712 x HTRI 105	F2:3	133	GW	2	10	[60]
Kitanokaori x Ldn/KU-2097	F2	132	DSA, DSH, DSM	1	3	[61]
PDW 233 x Bhalegaon 4	RIL	140	GY, NGS, GWS, GW	5	18	[62]
Rye selection 111 x Chinese spring	RIL	185	GW	8	17	[63]
Seri M82 x Babax	RIL	167	DSA, DSM, GY, NGm^2^, NDVI_g_, NDVI_v_, PH, GW	1	11	[64]
Chuanmai 42 x Chuannong 16 (F8)	RIL	127	GY, NGS, GWS, NGm^2^, NSm^2^, GW	7	35	[65]
MN98550 x MN99394	RIL	139	DSH, GY, LG, MG, PH, GD, GW, SG	4	41	[66]
Line 3228 x Jing 4839	F2:3	237	NFs, NGS, SL, NsS, GW	3	24	[67]
Arche x Récital	DH	222	NGm^2^, GW	31	42	[68]

^a^RIL: recombinant inbred line; DH: double haploid.

^b^For the extended name see list of acronyms.

^c^Number of QTLs subjected for projection onto the durum wheat consensus map.

**Fig 3:**
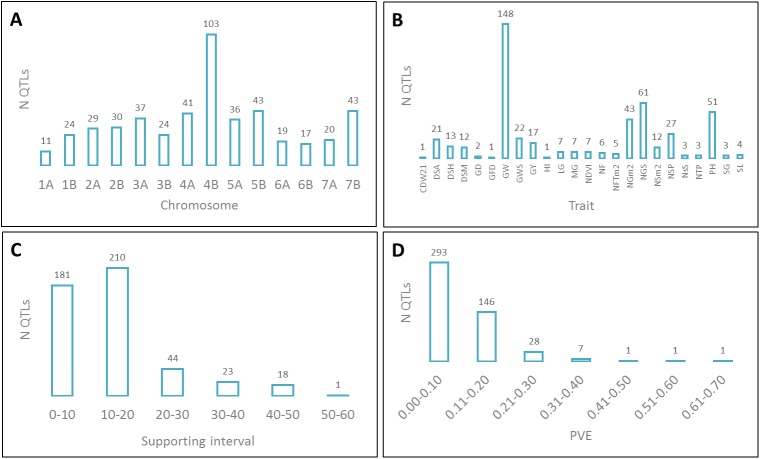
Parameters estimated in QTL studies collected for meta-analysis. Number of QTLs per A) chromosome, B) trait, C) supporting interval (cM) and D) phenotypic variance explained.

One hundred and ninety three QTLs (40%) were found in the A genome and 284 (60%) in the B genome. Chromosome 4B was the chromosome with the highest number of QTLs (103), whereas chromosome 1A was the one with the lowest (11) (Fig 3A). The number of QTLs per trait ranged from one for biomass, GFD and HI to 137 for GW. Grain weight traits (GWS, GW) included a total of 170 QTLs (36%) (Fig 3B). The distribution between the number of QTLs and size of the SI (Fig 3C) ranged from 1.9 to 51.9 cM with an average of 14.5 cM. Thirty-eight percent of the QTLs had a SI less than 10 cM and 82% of the QTLs less than 20 cM. The PVE explained by single QTL followed an L-shape distribution, with the majority of the QTLs (92%) showing a PVE < 0.2 (Fig 3D). PVE ranged from 0.03 to 0.68 with an average of 0.11.

The 477 QTLs projected onto the durum wheat consensus map were subjected to meta-analysis using the functions of Goffinet and Gerber [19] when the number of QTLs in a chromosome was lower than ten and those of Veyrieras [42] when the number was 10 or more. Following an AIC criterion, 409 QTLs were grouped into 71 MQTLs (S3 File). Thirteen QTLs remained as single QTLs clearly defined as not overlapping with other MQTLs and finally, 55 QTL SIs overlapped with different MQTLs and were not included in any of them based on the membership coefficient given by the software and if the peak of the QTL was located out of the MQTL SI.

The number of clustered QTLs ranged from two in 16 MQTLs on different chromosomes to 29 in MQTL40 on chromosome 4B, involving eight traits. The SI reported for the MQTLs ranged from 0.37 to 22.67 cM with an average of 5.44 cM. This means a reduction of more than 50% from those observed in the initial QTLs. The number of traits involved in each MQTL ranged from one in 14 MQTLs to eight in MQTL40 (S3 File).

Meta-QTLs for phenology and yield and components reported in previous studies [24, 25] were also projected onto the consensus map developed by Maccaferri [36] in order to find common regions with our analysis. Only MQTLs flanked by common markers among the original maps and [36] were projected (S4 File). Based on overlapping MQTL SIs, three MQTLs were found in common locations with those reported by [25] on chromosomes 2B, 5B and 7B, and 12 were in common with those reported by [24] located on chromosomes 1A, 2A, 3A, 4A, 4B, 5A, 6A and 7A (Fig 1, S3 and S4 Files).

## Discussion

### Linkage disequilibrium in Mediterranean landraces

It is well known that population structure influences linkage disequilibrium due to the presence of population stratification and an unequal distribution of alleles within groups, which can result in spurious associations [69]. The durum wheat Mediterranean landraces used in the present study showed a strong structural pattern [5], showing an eastern-western geographical pattern in agreement with dispersal of wheat across the Mediterranean basin [70, 71]. The mean *r*^*2*^ calculated for both inter- and intra-chromosomal loci was 0.02 and the extent of LD was estimated to be up to *ca*. 8 cM. These results were similar to the average of those reported by other authors using different types of variety panels. Maccaferri et al. [72], using a durum wheat panel representative of the major gene pools around the world and a set of 58 SSR markers, found high LD for inter-marker distance to be lower than 20 cM. Although a substantial reduction of spurious associations was obtained due to population structure, the authors concluded that the small number of accessions in each one of the subpopulations could lead a reduction in the power of the LD test. More recently, the same authors, using more SSR markers (179) in a collection of elite durum wheat accessions from the Mediterranean countries, South-Western USA and Mexico, found 27.8% of the loci in LD at *P*<0.01 and 15.1% at *P*<0.001, with a decay of the LD up to 10 cM, suggesting that for an adequate genome coverage, thousands of markers would be needed [6]. In a further study, [36] using the same panel of durum wheat genotypes but including a total of 2575 markers (SSR and DArT), reduced the extent of LD to 0 to 5 cM. In particular, the elite Mediterranean cultivars showed a LD decay within 2.20 cM. Somers et al. [73] found that 47.9% and 14.0% of locus comparisons showed significant LD at *P*>0.001 for bread and durum wheat, respectively, and LD decay over a distance between 0.5 and 5 cM for both wheat species. The collections studied showed a very high number of loci with rare alleles and, as reported by [74], the calculation of LD using *r*^*2*^ is dependent on allele frequency. The lower number of locus pairs in LD found in the durum collection was attributed by the authors to the diverse geographic origins and independent breeding programs for the durum entries. Laidò et al. [9], using a panel of 128 durum wheat accessions representing the Italian breeding programs over the last 100 years and 102 wild and domesticated accessions, found an average *r*^*2*^ for the whole collection of 0.02 and 22.7% of the markers in LD at *P*<0.01 with an extent of LD less than 14 cM, whereas LD decay for the durum sub-sample was estimated for a distance up to 18 cM. The pattern of LD is extremely population-dependent and related to the process of domestication. The authors explained the higher level of LD found in the durum subsample compared to the wild and domesticated accessions by the different levels of historical recombinations in the two samples. Hu et al. [13] with a panel of durum accessions of a worldwide origin observed that LD decay varied between chromosomes.

Similar results were reported by [13] and previously by [75] for chromosome 4B, being the second one with the lowest LD. The low LD found on 4B was explained by these authors as due to the selection for genes located in this chromosome. The use of a well-structured collection of accessions and the use of a large number of markers have led to a low percentage of locus pairs showing LD (15.1% at *P*<0.01 and 7.1% at *P*<0.001) and LD decay below 8 cM. The LD observed in our study also varies between chromosomes, and this will affect the resolution for association mapping.

### Marker trait associations

This study represents the first attempt to dissect the genetic basis of complex traits in a collection of Mediterranean durum wheat landraces. The collection was grown under the warm and dry conditions that characterize the western area of the Mediterranean basin [34]. A total of 245 associations between 1,072 markers and 13 traits were detected during three years in two locations of contrasting latitude in Spain. According to [9] MTAs located within map intervals of 5–10 cM are to be considered as belonging to the same QTL. Thus, in the current study, 53 regions across the durum wheat genome represent candidate QTLs. Twelve of these regions represented stable MTAs with reproducible associations in at least three environments.

Yield is the most important and genetically complex trait in wheat, being controlled by a large number of small effect QTLs and its dissection into components helps to identify genomic regions involved in yield formation. This complexity is revealed by the presence of MTAs for yield and components in all chromosome groups. In terms of stability across environments, only one region on chromosome 4B was detected for grain yield and two for grain weight (3B, 4B).

Grain number and grain weight are the main components affecting grain yield, and are usually negatively correlated [76]. As reported by [34] and [5], landraces from the East of the Mediterranean basin had a higher number of grains than those from the western area, with the opposite results for grain weight. Thus, detecting significant MTAs with a positive effect for both traits is challenging for the objective of increasing grain weight without reducing grain number. In our study, all but one of the MTAs for these traits were independent from each other. Only chromosome 1B MTAs for both traits were closely linked and as expected with opposite effects, positive for grain weight and negative for grain number.

Three loci for heading date on chromosomes 3B and 6B, reported by [36] were also identified in the current study for DBA and DSB, with one in 6B corresponding with a stable MTA region. On chromosome 2B, two MTAs for GFD and one for DSB were located close to *Ppd-B1* gene as reported by [25]. Additionally, genetic control of vernalization and earliness per se was reported in group 3 of chromosomes by [77].

Markers for PH were detected in chromosomes 1B, 2A, 2B, 4A, 4B, 5A, 6B and 7B. The locus on chromosome 2B may correspond with *Rht4* [78], whereas the stable MTAs on 4B and 7B with *Rht-B1* [79] and *Rht13* [80], respectively. MTAs for grain number and yield were located at the same locus on 7B suggesting that plant height genes could have pleiotropic effects on agronomic performance, as reported previously [80–82]. Biomass traits have not been studied in depth as they have been negatively selected during the breeding process to increase grain yield in elite cultivars. Biomass traits are generally undesirable, as they are inversely related to yield in determining the harvest index [83]. MTAs for biomass (CDW) were found widely distributed along the genome. Only two stable MTAs were found on chromosomes 6B and 7B.

### QTL meta-analysis

From a breeding perspective, QTL meta-analysis could gain power and precision if raw genotypic and phenotypic data from published QTL experiments are available [84]. As described by [85] this approach will help breeders and scientists to prioritize the selection of loci for breeding programs and for QTL cloning.

Several studies have identified QTLs controlling traits related to wheat yield and phenology development (for references see: [24, 25] and studies analysed in this study). QTL meta-analysis helps to integrate the QTL information available in order to determine regions of the genome that are frequently involved in trait variation and to narrow down the SI of QTLs. The results of QTL meta-analysis are strongly dependent on the precision of the initial QTL mapping, SI and projection quality [19]. In the current study, we only used QTLs fulfilling the requirements for QTL projection following the homothetic approach of Chardon et al. [39] and the BioMercator v4.2 software. QTL data from 26 independent studies were collected. Then, a QTL meta-analysis approach was carried out to detect genomic regions involved in yield, phenology and biomass traits previously identified.

QTL meta-analysis performed in this study revealed the presence of 84 (71 MQTL + 13 singletons) genomic regions involved in yield, yield components, and phenological development. Meta-analysis allowed for a remarkable simplification of the QTL regions, since the number was 6-fold fewer than the initial number of QTLs. MQTL positions reported in this study have some congruency with other results recently published. Three MQTLs were shared with [25] and 12 with [24] after projection of MQTL of those authors on the consensus map [36]. QTL data compilation showed that all wheat homologous groups of chromosomes are involved in the genetic control of yield, its related traits and biomass, whereas group 6 chromosomes lacked phenology QTLs.

Chromosomes 1B, 2B, 3A, 3B, 4A, 5A, 5B, 7A and 7B reported MQTLs for phenology traits. No MQTLs were detected on chromosome 2A where the photoperiod sensitivity gene *Ppd-A1* is located. On 2B two MQTLs were found, but their position did not correspond with *Ppd-B1*. These observations would suggest that there is little or no variation for *Ppd* genes in this collection of genotypes. Three MQTLs were detected on chromosome 5A from 34 to 104cM, from them MQTL44 and 45 could be related to a photoperiod sensitivity QTL reported by [86] and the vernalization gene *Vrn-A1*, respectively. Chromosome 5B presented three MQTLs in a position from 54 to 100cM. In this case MQTL50 could be related to *Vrn-B1*, whereas MQTL48 could be associated with intrinsic earliness or earliness *per se* (*eps*) QTL detected by [86]. Chromosomes 4A and 7B reported four and five MQTLs, respectively in the locations 60cM (4B) and 70cM (7B) suggesting important new regions controlling earliness. In addition, several QTLs were identified on chromosomes 1B, 3A, 3B, and 7A suggesting other regions with minor effects on earliness.

Grain yield can be partitioned into three major components: number of spikes per unit area, number of grains per spike and grain weight. These yield components are sequentially formed and are affected by other traits such as plant height, crop phenology and biomass. Together these traits make up grain yield, a complex trait controlled by multiple loci. However, some of these components are more stably inherited, such as grain weight [4, 87]. Candidate genes for grain weight have been identified in bread wheat in recent years. The sucrose synthase gene *TaSus2*, an ortholog of the maize gene *SuSy* [88], was isolated and mapped in wheat by [89]. The gene was mapped on chromosome 2B in a region where two MQTLs (MQTL15 and 17) were found in our study including QTLs for grain weight. The cell wall invertase (CWI) is a critical enzyme for sink tissue development and carbon partition, and has a high association with grain weight [90]. These authors characterized the full-length genomic DNA sequence of a *Cwi* gene located on wheat chromosome 2A, designated *TaCwi-A1*. MQTL11, including QTLs for TKW, is located in the vicinity of this gene. Rustgi et al. [91], from a search of candidate genes in rice, determined the location of two orthologous sequences underlying yield QTLs on chromosome 3A of wheat, *CKX2* and *GID2-like*. Location of these two genes may correspond with MQTL22, 23 and 24 but only the last two were associated with QTL involved in grain yield, grain weight and spikes per square meter. In rice, *OsGW2* encodes a RING-type E3 ubiquitin ligase and functions as a negative regulator of grain width and weight [92]. More recently, Simmonds et al. [93] positioned the wheat orthologous *TaGW2* on chromosome 6A. Opposite results found by other authors, i.e. a positive regulation of grain size [94] and a negative regulation [95], did not allow to conclude the exact effect of the gene on grain size and weight. In the present study MQTL54 and 55 were located within the QTL region reported by [93]. In rice, the *TGW6* gene determines grain weight and encodes a protein with indole-3-acetic acid (IAA)-glucose hydrolase activity [96]. Its ortholog in wheat, *TaTGW6*, is considered as a candidate gene related to grain development [97]. The gene was located on chromosome 4A, but no MQTLs for grain weight were found at that location in this study indicating that there is little or no variation for that gene in this collection of genotypes.

The meta-QTL analysis allowed us the identification of previously detected genomic regions harbouring QTLs for yield, phenology and biomass in durum wheat. In combination with the association mapping study reported in this work, they allowed us to dissect the genetic architecture of important traits for durum wheat breeding in Mediterranean landraces. Future studies using marker sequence and the recently updated wheat genome sequence assembly will be useful for searching and identifying putative candidate genes controlling the analysed traits.

## Supporting information

S1 File Linkage disequilibrium decay. Plots for LD decay in each one of the linkage groups.
(TIF)

S2 File Marker trait associations. Significant marker trait associations (MTAs) at–log_10_(*P*) > 3. Chr: chromosome. Position in the chromosome is expressed in cM (except for DArTs marked with ^*^ that is reported in Mb). NA: not assigned to any chromosome. U: unmapped. N: north. S: south, followed by a figure indicating the harvesting year M: mean value across the 3 years.
(DOCX)

S3 File Summary of MQTL information. It is reported the chromosome, the meta-QTL, its position in the consensus map [36] with a 95% of supporting interval (SI), the number of QTLs belonging to the MQTL, and finally the different traits associated to the MQTL.
(DOCX)

S4 File Chromosome position of previously mapped MQTLs. MQTLs from previous studies [4, 25] have been projected in the consensus map [36] for comparison with MTAs and MQTL reported in the present work.
(DOCX)

## Abbreviations

AM: association mapping
BP: Before present
CDW: crop dry weight at tillering (CDW_21_), jointing (CDW_33_), anthesis (CDW_65_), and physiological maturity (CDW_87_)
DBA: number of days from booting to anthesis
DSB: number of days from sowing to booting
GD: grain diameter
GFD: grain filling duration
GW: grain weight
GWS: grain weight per spike
GY: grain yield
HI: harvest index
LD: linkage disequilibrium
LG: percentage of large grains
MG: percentage of medium grains
MQTL: meta-QTL
MTA: marker trait associations
NDVI: normalized difference vegetation index at vegetative (NDVI_v_) and grain filling (NDVI_g_) stages
NFs: number of florets per spikelet
NFTm^2^: number of fertile tillers per square meter
NGm^2^: number of grains per square meter
NGS: number of grains per spike
NSm^2^: number of spikes per square meter
NSP: number of spikes per plant
NsS: number of spikelets per spike
NTP: number of tillers per plant
PH: plant height
QTL: quantitative trait locus
SG: percentage of small grains
SL: spike length
