# Supplementary figures and images for: Dissecting the old Mediterranean durum wheat genetic architecture for phenology, biomass and yield formation by association mapping and QTL meta-analysis
Source: PLoS One. 2017 May 25;12(5):e0178290. doi: 10.1371/journal.pone.0178290 (PMC5444813; doi:10.1371/journal.pone.0178290)

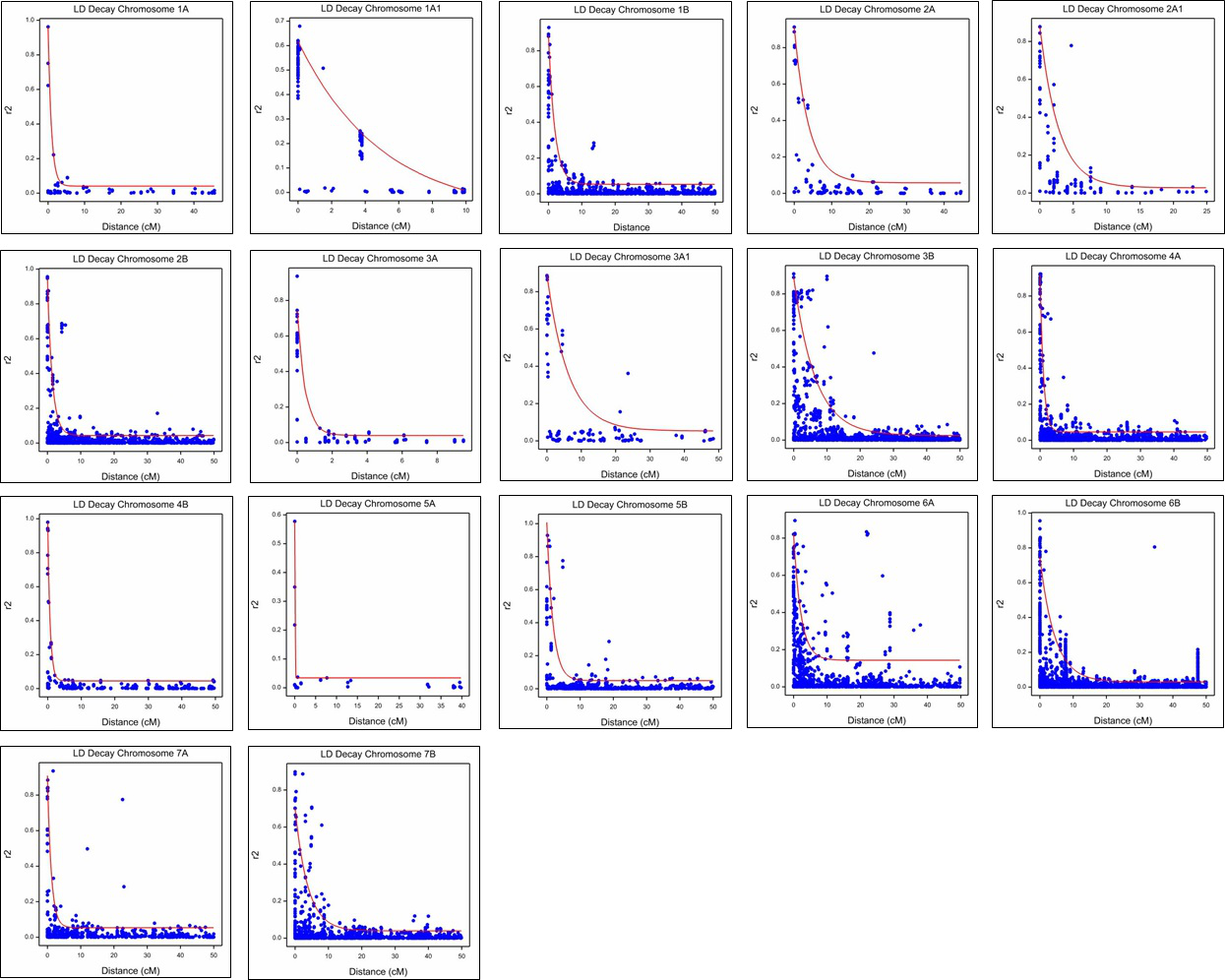

Supplement: S1 File — Plots for LD decay in each one of the linkage groups. (TIF) [file pone.0178290.s001.tif]
