# Supplementary material for: Dissecting the old Mediterranean durum wheat genetic architecture for phenology, biomass and yield formation by association mapping and QTL meta-analysis
Source: PLoS One. 2017 May 25;12(5):e0178290. doi: 10.1371/journal.pone.0178290 (PMC5444813; doi:10.1371/journal.pone.0178290)
Supplement: S2 File — Significant marker trait associations (MTAs) at–log10(P) > 3. Chr: chromosome. Position in the chromosome is expressed in cM (except for DArTs marked with * that is reported in Mb). NA: not assigned to any chromosome. U: unmapped. N: north. S: south, followed by a figure indicating the harvesting year M: mean value across the 3 years. (DOCX) [file pone.0178290.s002.docx]

**S2 File. Marker Trait Associations.**

| **Environment** | **Trait^a^** | **Marker^b^** | **Chr** | **Position** | **-log10(*P*)** | **Effect^c^** |
| --- | --- | --- | --- | --- | --- | --- |
| N7 | GY | wPt-9859 | 2B | 10.2 | 4.2 | -225.8 |
|  |  | wPt-8892 | 4B | 15.7 | 3.7 | -330.1 |
|  |  | wPt-6123 | 4B | 16.2 | 4.5 | -263.6 |
|  |  | wPt-5497 | 4B | 16.3 | 4.5 | -263.5 |
|  | NSm^2^ | wPt-0052 | 6B | 60.3 | 3.9 | 50.2 |
|  | NGm^2^ | wPt-2389 | 1B | 42.9 | 3.1 | -355.4 |
|  |  | wPt-6842 | 7A | 138.6 | 3.2 | -383.9 |
|  |  | wPt-2356 | 7B | 160.7 | 4.3 | 373.0 |
|  | GW | wPt-8892 | 4B | 15.7 | 5.5 | -4.4 |
|  |  | wPt-6123 | 4B | 16.2 | 4.0 | -3.0 |
|  |  | wPt-5497 | 4B | 16.3 | 4.0 | -3.0 |
|  | HI | wPt-6522 | 2B | 219 | 3.4 | 0.01 |
|  | DSB | wPt-5367 | 1A-LG2 | 26.4 | 3.5 | 1.3 |
|  |  | wPt-7320 | 2B | 47.7 | 3.8 | -1.1 |
|  |  | wPt-5990 | 4B | 72 | 3.9 | 1.4 |
|  |  | wPt-5803 | 5B | 131.1 | 4.0 | 1.3 |
|  |  | wPt-3304 | 6B | 5.1 | 3.9 | -1.5 |
|  |  | wPt-1852 | 6B | 5.1 | 3.8 | -1.4 |
|  | DBA | wPt-9859 | 2B | 10.2 | 3.4 | -0.65 |
|  |  | wPt-6174^*^ | 2B | 159.4 | 3.2 | -0.83 |
|  |  | wPt-8548 | 2B | U | 3.1 | -0.78 |
|  |  | wPt-9059 | 4A | 136.8 | 3 | -0.64 |
|  | GFD | wPt-9859 | 2B | 10.2 | 3.1 | 0.83 |
|  |  | wPt-5749 | 4A | 110.7 | 3.9 | 0.92 |
|  |  | wPt-3766 | 4A | 110.7 | 3.7 | 0.9 |
|  |  | wPt-0105 | 4A | 110.7 | 3.4 | 0.86 |
|  |  | wPt-6214 | 4A | 110.7 | 3.4 | 0.84 |
|  |  | wPt-9833 | 4A | 110.7 | 3.3 | 0.86 |
|  | CDW_21_ | wPt-1420 | 5B | 15.4 | 4.1 | 6.2 |
|  |  | wPt-9666 | 5B | 15.7 | 4.6 | 6.6 |
|  |  | wPt-9724 | 5B | 16.3 | 4.6 | 6.6 |
|  |  | wPt-8393^*^ | 5B | 532.1 | 3.3 | 6.5 |
|  |  | wPt-7954 | 6B | 23.6 | 3.2 | 5.1 |
|  |  | wPt-1163 | 7A | 60.8 | 3.7 | -6.9 |
|  | CDW_33_ | wPt-7502 | 3B | 124.9 | 3.4 | 16.4 |
|  | CDW_87_ | wPt-2866 | 3A-LG1 | U | 3.2 | 111.8 |
|  |  | wPt-1241 | 6B | 35.8 | 3.5 | -96.7 |
|  |  | tPt-5631 | NA | U | 3.3 | 89.1 |
|  | PH | wPt-6969^*^ | 2A | 687.5 | 3.2 | 8.6 |
|  |  | wPt-1140 | 2B | 133.4 | 3.2 | 10.0 |
|  |  | wPt-5135 | 5A | 148.4 | 3.2 | -7.8 |
|  |  | wPt-5343 | 7B | 152.2 | 3.4 | -5.5 |
|  |  | wPt-8615 | 7B | 152.2 | 3.0 | -5.1 |
|  |  | wPt-2491 | 3B | 181.6 | 3.6 | -1.6 |
| N8 | GY | wPt-5092 | 7A | 2.2 | 3.3 | 232.1 |
|  | NSm^2^ | wPt-7233 | 4B | -32.5 | 4.1 | -32.7 |
|  | NGm^2^ | wPt-5092 | 7A | 2.2 | 3.2 | 326.1 |
|  | GW | wPt-9859 | 2B | 10.2 | 3.2 | -1.9 |
|  |  | wPt-2293 | 2B | 145.9 | 3.3 | 1.8 |
|  |  | wPt-0990 | 3B | 184.4 | 3.0 | 1.9 |
|  |  | wPt-7418^*^ | 3B | 792.7 | 3.7 | -1.9 |
|  |  | wPt-8892 | 4B | 15.7 | 3.1 | -2.9 |
|  |  | wPt-5118^*^ | 5B | 697.6 | 4.5 | -3.2 |
|  |  | wPt-5256 | 6B | 36.6 | 3.3 | -2.0 |
|  |  | wPt-9952^*^ | 6B | 715.0 | 3.5 | 1.6 |
|  | HI | wPt-2938 | 3A-LG1 | 9.5 | 3.1 | -0.01 |
|  | DSB | wPt-6709 | 1A-LG1 | 4.5 | 3.4 | 0.9 |
|  |  | wPt-8172 | 1A-LG1 | U | 3.4 | -1.0 |
|  |  | wPt-1852 | 6B | 5.1 | 3.8 | -1.1 |
|  |  | wPt-3304 | 6B | 5.1 | 3.7 | -1.1 |
|  |  | wPt-7846 | 6B | 60.3 | 4.0 | -1.4 |
|  |  | rPt-9611 | NA | U | 5.2 | -1.4 |
|  | DBA | wPt-6692 | 5B | 25.6 | 3.1 | -0.47 |
|  | GFD | wPt-5513 | 2B | 45.2 | 3.2 | 0.84 |
|  |  | wPt-5788 | 2B | 45.3 | 3 | 0.81 |
|  |  | wPt-0980 | 7B | 117.6 | 3.1 | 1.12 |
|  | CDW_65_ | wPt-9320 | 2A-LG1 | 90.3 | 3.5 | -85.1 |
|  |  | wPt-3730 | 7B | 104.7 | 3.4 | -88.4 |
|  | PH | wPt-8172 | 1A-LG1 | U | 3.8 | -4.6 |
|  |  | wPt-1684 | 1B | 19.2 | 3.1 | 3.9 |
|  |  | wPt-1140 | 2B | 133.4 | 3.9 | 9.8 |
|  |  | wPt-6209 | 4B | 50 | 3.5 | 3.9 |
|  |  | wPt-2095 | 6B | 24.5 | 3.2 | 3.9 |
|  |  | wPt-1437 | 6B | 24.7 | 3.3 | 4.1 |
|  |  | wPt-2162 | 6B | 158 | 4.0 | -4.2 |
|  |  | wPt-5343 | 7B | 152.2 | 4.1 | -5.5 |
|  |  | wPt-8615 | 7B | 152.2 | 4.0 | -5.4 |
| N9 | GY | wPt-2314 | 2B | 59.6 | 3.2 | -187.1 |
|  |  | wPt-6047 | 3B | 97.1 | 3.8 | -211.5 |
|  | NSm^2^ | wPt-5395 | 6A | 0 | 3.1 | -32.4 |
|  |  | wPt-5633 | 6A | 0.4 | 3.8 | -37.1 |
|  | GW | wPt-0990 | 3B | 184.4 | 4.3 | 1.9 |
|  |  | wPt-6000^*^ | 3B | 791.6 | 3.4 | 1.7 |
|  | DSB | wPt-8320 | 1B | U | 3.1 | 1.6 |
|  |  | wPt-0707^#^ | 5A | U | 4.1 | 1.6 |
|  | GFD | wPt-9316 | 2B | 61.7 | 4.3 | -0.64 |
|  |  | wPt-0724 | 2B | 61.9 | 3.9 | -0.59 |
|  |  | tPt-4209 | 6A | 95.3 | 3.9 | -0.62 |
|  |  | tPt-4216 | NA | U | 3.8 | 0.69 |
|  |  | rPt-0699 | NA | U | 3.2 | 0.55 |
|  | CDW_21_ | tPt-6661 | 6A | 143 | 3.2 | 6.0 |
|  | CDW_65_ | wPt-5108^*^ | 2B | 791.4 | 4.0 | -102.2 |
|  | CDW_87_ | wPt-1163 | 7A | 60.8 | 3.6 | 74.8 |
|  | PH | wPt-8172 | 1A-LG1 | U | 3.3 | -3.2 |
|  |  | wPt-8838 | 1A-LG1 | U | 3.4 | -3.4 |
|  |  | wPt-6209 | 4B | 50 | 3.2 | 2.8 |
| NM | GY | wPt-9859 | 2B | 10.2 | 3.3 | -152.5 |
|  |  | wPt-6123 | 4B | 16.2 | 3.3 | -185.7 |
|  |  | wPt-5497 | 4B | 16.3 | 3.3 | -186.6 |
|  |  | wPt-2356 | 7B | 160.7 | 3.2 | 114.0 |
|  | NSm^2^ | wPt-1377 | 6A | 0.2 | 3.6 | 22.5 |
|  |  | wPt-5633 | 6A | 0.4 | 4.1 | -25.0 |
|  | NGm^2^ | wPt-2356 | 7B | 160.7 | 3.4 | 262.5 |
|  | GW | wPt-3451 | 1B | 43.9 | 3.0 | 1.5 |
|  |  | wPt-0990 | 3B | 184.4 | 3.6 | 1.7 |
|  |  | wPt-6000^*^ | 3B | 791.6 | 3.5 | 1.6 |
|  |  | wPt-8892 | 4B | 15.7 | 4.3 | -2.8 |
|  |  | wPt-6123 | 4B | 16.2 | 3.3 | -1.9 |
|  |  | wPt-5497 | 4B | 16.3 | 3.3 | -1.9 |
|  |  | wPt-5118^*^ | 5B | 697.6 | 3.6 | -2.3 |
|  | HI | wPt-0049 | 2B | 219.8 | 3.4 | -0.01 |
|  |  | rPt-7987 | 4A | U | 3.2 | -0.01 |
|  |  | wPt-7763 | 7A | 221 | 3.3 | -0.01 |
|  |  | wPt-5138 | 7B | 189 | 3.6 | -0.01 |
|  |  | tPt-6363 | 7B | 197.9 | 3.1 | -0.01 |
|  | DSB | wPt-5432 | 3B | 37.2 | 3.8 | 1.1 |
|  |  | wPt-0707^#^ | 5A | U | 3.6 | 1.3 |
|  |  | wPt-5803 | 5B | 131.1 | 4.0 | 0.9 |
|  |  | rPt-9065 | 6A | 28 | 3.2 | -0.9 |
|  |  | wPt-1852 | 6B | 5.1 | 3.9 | -1.0 |
|  |  | wPt-3304 | 6B | 5.1 | 4.1 | -1.0 |
|  |  | wPt-7846 | 6B | 60.3 | 3.5 | -1.2 |
|  |  | rPt-9611 | NA | U | 3.5 | -1.0 |
|  | DBA | wPt-6692 | 5B | 25.6 | 3.2 | -0.38 |
|  | GFD | wPt-9859 | 2B | 10.2 | 4.4 | 0.7 |
|  |  | wPt-5749 | 4A | 110.7 | 3.4 | 0.61 |
|  |  | wPt-3766 | 4A | 110.7 | 3.1 | 0.58 |
|  |  | wPt-9833 | 4A | 110.7 | 3.1 | 0.59 |
|  |  | wPt-7975 | 7B | 3.6 | 3.3 | 1 |
|  |  | wPt-5846 | 7B | 3.6 | 3.2 | 1 |
|  | CDW_21_ | wPt-5497 | 4B | 16.3 | 3.0 | -5.3 |
|  |  | tPt-6363 | 7B | 197.9 | 3.3 | 5.3 |
|  | CDW_33_ | wPt-7502 | 3B | 124.9 | 3.2 | 13.7 |
|  | CDW_65_ | wPt-9320 | 2A-LG1 | 90.3 | 3.2 | -51.8 |
|  |  | wPt-9859 | 2B | 10.2 | 3.2 | -60.7 |
|  |  | wPt-8615 | 7B | 152.2 | 3.1 | -62.5 |
|  | PH | wPt-8172 | 1A-LG1 | U | 3.3 | -3.2 |
|  |  | wPt-8838 | 1A-LG1 | U | 4.2 | -3.5 |
|  |  | wPt-9859 | 2B | 10.2 | 3.1 | -3.3 |
|  |  | wPt-6209 | 4B | 50 | 3.6 | 3.0 |
|  |  | wPt-5138 | 7B | 189 | 3.0 | 3.1 |
| S7 | GY | wPt-3037 | 2A-LG1 | 97.6 | 3.2 | -236.2 |
|  |  | wPt-2435 | 2A-LG1 | 100 | 3.3 | -249.1 |
|  |  | wPt-8686 | 3B | 47.9 | 3.4 | 250.0 |
|  |  | wPt-1349 | 3B | 47.9 | 3.4 | 251.6 |
|  | NSm^2^ | wPt-7649 | 2A-LG2 | 18.2 | 3.1 | 30.5 |
|  | NGm^2^ | wPt-3037 | 2A-LG1 | 97.6 | 3.1 | -439.7 |
|  |  | wPt-2435 | 2A-LG1 | 100 | 3.4 | -485.1 |
|  |  | wPt-9302 | 2A-LG2 | 16.7 | 3.3 | 659.9 |
|  | GW | wPt-4676 | 1A-LG1 | U | 4.1 | 2.8 |
|  |  | wPt-0990 | 3B | 184.4 | 5.4 | 3.1 |
|  |  | wPt-6000^*^ | 3B | 791.6 | 5.0 | 3.1 |
|  | DSB | wPt-9679 | 6A | 26.6 | 3.4 | -0.8 |
|  |  | wPt-5652 | 6A | 27.7 | 3.3 | -0.9 |
|  |  | wPt-7027 | 6A | 28 | 3.1 | -0.7 |
|  |  | wPt-11589 | 6B | 83.6 | 4.6 | -1.0 |
|  | DBA | wPt-4205^#^ | 7A | U | 3.2 | 0.75 |
|  | CDW_21_ | wPt-6882 | 1A-LG1 | 12.4 | 3.1 | -24.0 |
|  |  | wPt-1601 | 2B | 7.6 | 3.1 | 25.6 |
|  | CDW_33_ | wPt-8976 | 6B | 86.6 | 3.5 | -67.4 |
|  | CDW_65_ | wPt-7649 | 2A-LG2 | 18.2 | 3.8 | 112.5 |
|  |  | wPt-8976 | 6B | 86.6 | 3.5 | -77.9 |
|  |  | wPt-1364 | 6B | 86.6 | 3.3 | 76.1 |
|  | PH | wPt-8267 | 1B | U | 3.8 | -4.5 |
|  |  | wPt-9859 | 2B | 10.2 | 6.1 | -6.8 |
|  |  | wPt-2162 | 6B | 158 | 3.2 | -3.8 |
|  |  | wPt-5343 | 7B | 152.2 | 3.3 | -5.2 |
|  |  | wPt-8615 | 7B | 152.2 | 3.1 | -5.0 |
| S8 | GY | wPt-8393^*^ | 5B | 532.1 | 3.1 | -324.1 |
|  | NSm^2^ | wPt-4537 | 4B | 0 | 3.4 | 36.8 |
|  | NGm^2^ | wPt-3198 | 1A-LG1 | 4.5 | 3.0 | -546.6 |
|  |  | wPt-0841 | 7B | 116.3 | 3.0 | 646.7 |
|  | HI | wPt-6854 | 3A-LG1 | 6.2 | 3.4 | 0.02 |
|  | DBA | wPt-1091 | 4A | 93 | 3.5 | 1.02 |
|  |  | wPt-6728 | 4A | 93.7 | 3.3 | 0.95 |
|  | GFD | wPt-0983 | 1B | -7.9 | 3.5 | 0.82 |
|  |  | wPt-9223 | 4B | 65.8 | 3.5 | 0.76 |
|  |  | wPt-11493 | 4B | 65.8 | 3.5 | 0.75 |
|  |  | wPt-3255 | 4B | 65.8 | 3.3 | 0.74 |
|  | CDW_33_ | wPt-2151 | 4A | 138.4 | 3.9 | -105.5 |
|  | CDW_65_ | tPt-9048 | 6B | 158 | 3.1 | 137.0 |
|  | CDW_87_ | wPt-4537 | 4B | 0 | 3.9 | 145.5 |
|  | PH | wPt-2696 | 2A-LG2 | 22 | 3.2 | -6.4 |
|  |  | wPt-7939 | 4A | 93.3 | 3.4 | -4.7 |
|  |  | wPt-5343 | 7B | 152.2 | 3.6 | -6.1 |
|  |  | wPt-8615 | 7B | 152.2 | 3.2 | -5.6 |
| S9 | NSm^2^ | tPt-1772^*^ | 1B | 13.4 | 3.8 | 14.9 |
|  |  | wPt-1912^*^ | 1B | 1.8 | 3.3 | 13.6 |
|  |  | wPt-1238 | 1B | U | 3.1 | 13.1 |
|  |  | wPt-3411 | 1B | U | 3.4 | 13.9 |
|  | NGm^2^ | wPt-2847 | 1A-LG2 | 24.3 | 3.6 | 633.3 |
|  |  | wPt-1159 | 3B | 76.5 | 3.2 | 576.1 |
|  |  | rPt-6847 | 4B | 60.1 | 3.2 | -395.3 |
|  | GW | wPt-3451 | 1B | 43.9 | 3.1 | 1.1 |
|  | DSB | tPt-0799^*^ | 2A | 29.5 | 3.0 | 1.1 |
|  |  | wPt-1140 | 2B | 133.4 | 3.1 | 2.0 |
|  |  | wPt-3725 | 3B | 162.4 | 3.7 | -1.5 |
|  | DBA | wPt-1374 | 1B | 26.2 | 3 | 0.8 |
|  |  | wPt-3651 | 2B | 150.6 | 3 | -0.74 |
|  | GFD | wPt-5346 | 5B | 18.5 | 3.5 | 1.2 |
|  |  | wPt-5175 | 5B | 18.7 | 3.6 | 1.2 |
|  | CDW_21_ | wPt-1725 | 6B | 16.5 | 3.9 | 17.3 |
|  |  | wPt-0073 | 6B | 16.5 | 3.6 | 16.8 |
|  | CDW_65_ | wPt-8267 | 1B | U | 5.1 | -44.2 |
|  |  | wPt-9859 | 2B | 10.2 | 4.0 | -45.6 |
|  |  | wPt-6854 | 3A-LG1 | 6.2 | 3.2 | -37.9 |
|  |  | wPt-7992 | 3A-LG1 | 6.2 | 3.1 | -36.5 |
|  |  | wPt-5343 | 7B | 152.2 | 4.9 | -53.9 |
|  |  | wPt-8615 | 7B | 152.2 | 4.5 | -50.6 |
|  | CDW_87_ | rPt-5396 | 3B | 78.6 | 3.0 | 77.8 |
|  |  | wPt-4127 | 3B | 102.7 | 3.0 | 77.8 |
|  |  | wPt-7412 | 4B | 72.3 | 3.2 | -67.1 |
|  |  | wPt-2431 | NA | U | 3.4 | -74.6 |
|  |  | wPt-6939 | NA | U | 4.2 | -81.3 |
|  | PH | wPt-8172 | 1A-LG1 | U | 3.9 | -4.6 |
|  |  | wPt-9859 | 2B | 10.2 | 4.1 | -5.1 |
|  |  | wPt-5343 | 7B | 152.2 | 4.0 | -5.4 |
|  |  | wPt-8615 | 7B | 152.2 | 3.2 | -4.7 |
| SM | NSm^2^ | wPt-5385 | 1B | 20.6 | 3.3 | 14.3 |
|  | GW | wPt-0011^*^ | 1A-LG1 | 532.2 | 3.1 | -1.4 |
|  |  | wPt-6530 | 1A-LG1 | U | 3.4 | -1.3 |
|  |  | wPt-8838 | 1A-LG1 | U | 3.2 | -1.3 |
|  |  | wPt-8882 | 1A-LG1 | U | 3.7 | -1.4 |
|  |  | wPt-4676 | 1A-LG1 | U | 3.6 | -1.3 |
|  |  | wPt-0990 | 3B | 184.4 | 3.3 | 1.4 |
|  | HI | wPt-0689 | 6A | -0.8 | 3.1 | 0.01 |
|  |  | wPt-3524 | 6A | -0.8 | 3.4 | 0.01 |
|  |  | rPt-9065 | 6A | 28 | 3.7 | 0.01 |
|  |  | wPt-6904 | 6A | 28 | 3.6 | 0.01 |
|  |  | tPt-2833 | 6A | 28 | 3.5 | 0.01 |
|  |  | tPt-0877 | 6A | 28 | 3.2 | 0.01 |
|  | DSB | wPt-6894 | 2B | 227.1 | 3.3 | -0.9 |
|  |  | wPt-3725 | 3B | 162.4 | 5.4 | -1.3 |
|  |  | wPt-11589 | 6B | 83.6 | 3.9 | -1.0 |
|  | DBA | wPt-0086 | 3B | 45.9 | 3.5 | 0.65 |
|  | CDW_21_ | wPt-1725 | 6B | 16.5 | 3.4 | 13.2 |
|  |  | wPt-0073 | 6B | 16.5 | 3.4 | 13.3 |
|  | CDW_33_ | wPt-0554 | 6B | 48 | 3.1 | 38.6 |
|  |  | wPt-1364 | 6B | 86.6 | 3.1 | 39.2 |
|  | CDW_65_ | tPt-9048 | 6B | 158 | 3.7 | 69.2 |
|  | PH | wPt-8172 | 1A-LG1 | U | 3.9 | -4.6 |
|  |  | wPt-9859 | 2B | 10.2 | 4.6 | -5.4 |
|  |  | wPt-5135 | 5A | 148.4 | 3.0 | -6.8 |
|  |  | wPt-5343 | 7B | 152.2 | 4.0 | -5.3 |
|  |  | wPt-8615 | 7B | 152.2 | 3.2 | -4.7 |

Significant marker trait associations (MTAs) at –log_10_(*P*) > 3. Chr: chromosome. Position in the chromosome is expressed in cM (except for DArTs marked with ^*^ that is reported in Mb). NA: not assigned to any chromosome. U: unmapped. N: north. S: south, followed by a figure indicating the harvesting year M: mean value across the 3 years.

^a^For the extended name see list of acronyms.

^b^DArTs marked with ^#^ represent markers which chromosome was identified using the ‘Wheat consensus map version 3.0’ and DArTs markerd with ^*^ represent markers which physical position was reported from the wheat genome sequence. The position reported for these markers is given in Mb.

^c^Minor allele effect.
