# Supplementary material for: Dissecting the old Mediterranean durum wheat genetic architecture for phenology, biomass and yield formation by association mapping and QTL meta-analysis
Source: PLoS One. 2017 May 25;12(5):e0178290. doi: 10.1371/journal.pone.0178290 (PMC5444813; doi:10.1371/journal.pone.0178290)
Supplement: S3 File — It is reported the chromosome, the meta-QTL, its position in the consensus map [36] with a 95% of supporting interval (SI), the number of QTLs belonging to the MQTL, and finally the different traits associated to the MQTL. (DOCX) [file pone.0178290.s003.docx]

**S3 File. Summary of MQTL information.**

| **Chromosome** | **MQTL** | **Position (cM)** | **SI (95%)** | **N QTLs** | **Traits^a^** |
| --- | --- | --- | --- | --- | --- |
| 1A (LG1) | 1 | 24.88 | 8.99 | 4 | PH, GW |
|  | 2 | 34.33 | 9.67 | 4 | PH, NsS, GW |
| 1B | 3 | 11.53 | 2.36 | 2 | DSH, GW |
|  | 4 | 23.69 | 4.01 | 4 | DSA, DSM |
|  | 5 | 48.89 | 6.68 | 4 | GY, NGS, GW |
|  | 6 | 77.68 | 6.47 | 2 | NGS |
|  | 7 | 106.99 | 6.01 | 5 | GY, NGm^2^ |
| 2A (LG1) | 8 | 0.54 | 3.71 | 4 | GW |
|  | 9 | 16.91 | 5.99 | 2 | GY |
|  | 10 | 38.66 | 6.89 | 2 | NDVI |
|  | 11 | 63.43 | 0.63 | 18 | LG, MG, PH, SGW, SG, GW |
| 2A (LG2) | 12 | 10 | 11.95 | 2 | NGS |
| 2B | 13 | 18.85 | 3.76 | 3 | DSM, GW |
|  | 14 | 37.18 | 2.59 | 6 | GWS, NSP, GW |
|  | 15 | 60.52 | 3.58 | 4 | GW |
|  | 16 | 69.37 | 5.51 | 2 | NSm^2^ |
|  | 17 | 77.85 | 5.65 | 2 | NGS, GW |
|  | 18 | 107.71 | 7.87 | 6 | NGS, NGm^2^ |
|  | 19 | 165.92 | 1.95 | 2 | DSH, NDVI |
| 3A (LG1) | 20 | 1.16 | 2.34 | 4 | NGS, PH, GW |
|  | 21 | 6.5 | 5.37 | 3 | PH, GW |
| 3A (LG2) | 22 | 10.93 | 3.69 | 2 | DSA, PH |
|  | 23 | 18.37 | 3.55 | 5 | GY, NSm^2^, PH |
|  | 24 | 26.12 | 6.31 | 2 | PH, GW |
|  | 25 | 39.22 | 4.07 | 7 | GY, NGS, PH |
|  | 26 | 54.18 | 7.74 | 2 | NGS |
|  | 27 | 69.14 | 3.87 | 7 | NGS, NSP, GW |
|  | 28 | 93.83 | 3.22 | 5 | GW, DSA, GY, NSm^2^, PH |
| 3B | 29 | 96.03 | 1.84 | 10 | GY, PH, GW |
|  | 30 | 120.5 | 2.9 | 3 | PH |
|  | 31 | 156.15 | 0.73 | 9 | CDW_21_, DSA, GY, NGm^2^, NSm^2^,GW |
| 4A | 32 | 56 | 8.51 | 17 | NFs, NGS,NGm^2^, PH, SL, GW |
|  | 33 | 75.27 | 8.06 | 2 | DSA |
|  | 34 | 105.76 | 15.83 | 2 | DSA, FTm^2^ |
|  | 35 | 127.34 | 12.44 | 10 | DSA, DSH, DSM, NGS |
|  | 36 | 133.37 | 9.51 | 8 | DSA, DSH, NGS, NSP |
| 4B | 37 | 2.87 | 3.41 | 4 | PH, GW |
|  | 38 | 15.16 | 5.25 | 2 | GW |
|  | 39 | 21.61 | 3.15 | 26 | NGS, GWS, NGm^2^, PH, NSP, GW |
|  | 40 | 37.54 | 6.87 | 29 | GW, GY, NGS, GWS, NGm^2^, PH, NSP, GW |
|  | 41 | 71.42 | 0.68 | 4 | NGm^2^, NSP, GW |
| 5A | 42 | 16.32 | 2.06 | 11 | GFD, NGS, NSP, NTP, GW |
|  | 43 | 33.99 | 9.85 | 2 | DSM, NGm^2^ |
|  | 44 | 74.97 | 2.85 | 14 | DSA, GWS, NGm^2^, GW |
|  | 45 | 103.9 | 6.15 | 5 | DSA, DSH, DSM, NGS, NGm^2^ |
|  | 46 | 160.1 | 22.67 | 4 | SL, NsS, GW |
| 5B | 47 | 45.35 | 2.63 | 15 | NGS, GWS, NGm^2^, GW |
|  | 48 | 54.21 | 4.98 | 4 | DSA, NSm^2^, GW |
|  | 49 | 68.96 | 3.31 | 7 | DSA, DSM, GY, NSm^2^, GW |
|  | 50 | 100.24 | 3.32 | 4 | DSH, HI, NSP |
|  | 51 | 118.74 | 2.85 | 3 | GWS, GW |
|  | 52 | 136.64 | 0.37 | 8 | NFs, FTm^2^, GWS, NSP, GW |
| 6A | 53 | 2.4 | 5.46 | 2 | NGS |
|  | 54 | 39.92 | 2.34 | 3 | LG, MG, GW |
|  | 55 | 80.95 | 7.17 | 4 | GY, GWS, GW |
|  | 56 | 131.39 | 1.03 | 8 | NDVI, GW |
| 6B | 57 | 60.53 | 3.75 | 13 | LG, MG, SGD, SGW, GW |
|  | 58 | 119.6 | 3.29 | 3 | NGm^2^, GW |
| 7A | 59 | 1.58 | 4.78 | 4 | GW |
|  | 60 | 18.8 | 10.29 | 3 | NGS, GW |
|  | 61 | 84.86 | 4.98 | 8 | NGS, GW |
|  | 62 | 127.85 | 8.03 | 4 | DSH, DSM, NGS |
| 7B | 63 | 2.35 | 3.2 | 2 | DSH, FTm^2^ |
|  | 64 | 17.42 | 3.9 | 7 | DSH, PH |
|  | 65 | 40.34 | 4.15 | 4 | DSA, PH |
|  | 66 | 54.42 | 2.89 | 3 | DSA |
|  | 67 | 73.31 | 3.76 | 3 | DSA, NGS, PH |
|  | 68 | 81.75 | 6.08 | 3 | GWS, NSP, GW |
|  | 69 | 93.4 | 2.88 | 5 | GWS, GW |
|  | 70 | 103.99 | 2.3 | 7 | NGS, GW |
|  | 71 | 183.55 | 2.61 | 5 | NGS, GW |

It is reported the chromosome, the meta-QTL, its position in the consensus map [36] with a 95% of supporting interval (SI), the number of QTLs belonging to the MQTL, and finally the different traits associated to the MQTL.

^a^For the extended name see list of acronyms.
