# Supplementary material for: Dissecting the old Mediterranean durum wheat genetic architecture for phenology, biomass and yield formation by association mapping and QTL meta-analysis
Source: PLoS One. 2017 May 25;12(5):e0178290. doi: 10.1371/journal.pone.0178290 (PMC5444813; doi:10.1371/journal.pone.0178290)
Supplement: S4 File — MQTLs from previous studies [4, 25] have been projected in the consensus map [36] for comparison with MTAs and MQTL reported in the present work. (DOCX) [file pone.0178290.s004.docx]

**S4 File. Chromosome position of previously mapped MQTLs.**

| **MQTL** | **Chromosome** | **Position (cM)** | **SI (95%)** |
| --- | --- | --- | --- |
| Hanocq_2007_MQTL2 | 2B | 57.78 | 6.6 |
| Hanocq_2007_MQTL3 | 2B | 131.00 | 13 |
| Hanocq_2007_MQTL7 | 4A | 43.86 | 4.8 |
| Hanocq_2007_MQTL8 | 4B | 63.98 | 1.1 |
| Hanocq_2007_MQTL9 | 5A | 86.11 | 7.7 |
| Hanocq_2007_MQTL10 | 5A | 125.80 | 3.2 |
| Hanocq_2007_MQTL11 | 5B | 30.14 | 16.9 |
| Hanocq_2007_MQTL12 | 5B | 40.91 | 9.8 |
| Hanocq_2007_MQTL13 | 5B | 112.57 | 4.2 |
| Hanocq_2007_MQTL15 | 6A | 33.39 | 4.5 |
| Hanocq_2007_MQTL16 | 7A | 98.34 | 18.7 |
| Hanocq_2007_MQTL17 | 7B | 30.80 | 21 |
| Zhang_2010_MQTL1 | 1A (LG1) | 22.94 | 5.99 |
| Zhang_2010_MQTL3 | 1A (LG2) | 38.58 | 3.35 |
| Zhang_2010_MQTL4 | 1B | 30.49 | 1.01 |
| Zhang_2010_MQTL5 | 1B | 0.98 | 1.63 |
| Zhang_2010_MQTL8 | 2A (LG1) | 2.22 | 0.96 |
| Zhang_2010_MQTL9 | 2A (LG1) | 49.95 | 3.33 |
| Zhang_2010_MQTL10 | 2A (LG1) | 71.73 | 10.63 |
| Zhang_2010_MQTL11 | 2B | 12.66 | 4.19 |
| Zhang_2010_MQTL12 | 2B | 140.61 | 1.52 |
| Zhang_2010_MQTL13 | 2B | 168.64 | 1.25 |
| Zhang_2010_MQTL21 | 3A (LG1) | 3.21 | 2.29 |
| Zhang_2010_MQTL23 | 3A (LG2) | 22.88 | 6.03 |
| Zhang_2010_MQTL24 | 3B | 69.56 | 0.91 |
| Zhang_2010_MQTL25 | 3B | 40.73 | 4.15 |
| Zhang_2010_MQTL26 | 3B | 90.75 | 1.85 |
| Zhang_2010_MQTL27 | 3B | 57.36 | 2.94 |
| Zhang_2010_MQTL28 | 3B | 135.58 | 4.04 |
| Zhang_2010_MQTL29 | 3B | 179.77 | 5.67 |
| Zhang_2010_MQTL30 | 4A | 12.61 | 2.19 |
| Zhang_2010_MQTL31 | 4A | 64.29 | 3.73 |
| Zhang_2010_MQTL32 | 4A | 58.65 | 0.69 |
| Zhang_2010_MQTL33 | 4B | 35.60 | 9.20 |
| Zhang_2010_MQTL34 | 4B | 73.56 | 5.57 |
| Zhang_2010_MQTL38 | 5A | 12.93 | 6.75 |
| Zhang_2010_MQTL39 | 5A | 17.71 | 3.01 |
| Zhang_2010_MQTL40 | 5A | 70.14 | 2.77 |
| Zhang_2010_MQTL41 | 5A | 105.78 | 1.79 |
| Zhang_2010_MQTL42 | 5A | 125.36 | 1.88 |
| Zhang_2010_MQTL43 | 5A | 143.70 | 1.31 |
| Zhang_2010_MQTL44 | 6A | 31.36 | 11.46 |
| Zhang_2010_MQTL45 | 6A | 49.74 | 13.24 |
| Zhang_2010_MQTL46 | 6A | 83.72 | 4.31 |
| Zhang_2010_MQTL47 | 6A | 96.29 | 2.11 |
| Zhang_2010_MQTL48 | 7A | 76.68 | 16.86 |
| Zhang_2010_MQTL49 | 7A | 94.17 | 10.56 |
| Zhang_2010_MQTL50 | 7A | 128.40 | 6.74 |
| Zhang_2010_MQTL51 | 7A | 196.86 | 3.99 |

MQTLs from previous studies [4, 25] have been projected in the consensus map [36] for comparison with MTAs and MQTL reported in the present work.
